# Evaluation of a highly refined prediction model in knowledge-based volumetric modulated arc therapy planning for cervical cancer

**DOI:** 10.1186/s13014-021-01783-9

**Published:** 2021-03-22

**Authors:** Mingli Wang, Huikuan Gu, Jiang Hu, Jian Liang, Sisi Xu, Zhenyu Qi

**Affiliations:** 1 Department of Radiation Oncology, Sun Yat-Sen University Cancer Center, Guangzhou, People’s Republic of China; 2 State Key Laboratory of Oncology in South China, Collaborative Innovation Center for Cancer Medicine, Guangzhou, People’s Republic of China; 3 Guangdong Key Laboratory of Nasopharyngeal Carcinoma Diagnosis and Therapy, Guangzhou, People’s Republic of China

**Keywords:** Prediction model, Volumetric modulated arc therapy, Knowledge-based planning, Progressive training, Cervical cancer

## Abstract

**Background and purpose:**

To explore whether a highly refined dose volume histograms (DVH) prediction model can improve the accuracy and reliability of knowledge-based volumetric modulated arc therapy (VMAT) planning for cervical cancer.

**Methods and materials:**

The proposed model underwent repeated refining through progressive training until the training samples increased from initial 25 prior plans up to 100 cases. The estimated DVHs derived from the prediction models of different runs of training were compared in 35 new cervical cancer patients to analyze the effect of such an interactive plan and model evolution method. The reliability and efficiency of knowledge-based planning (KBP) using this highly refined model in improving the consistency and quality of the VMAT plans were also evaluated.

**Results:**

The prediction ability was reinforced with the increased number of refinements in terms of normal tissue sparing. With enhanced prediction accuracy, more than 60% of automatic plan-6 (AP-6) plans (22/35) can be directly approved for clinical treatment without any manual revision. The plan quality scores for clinically approved plans (CPs) and manual plans (MPs) were on average 89.02 ± 4.83 and 86.48 ± 3.92 (*p* < 0.001). Knowledge-based planning significantly reduced the D_mean_ and V_18 Gy_ for kidney (L/R), the D_mean_, V_30 Gy_, and V_40 Gy_ for bladder, rectum, and femoral head (L/R).

**Conclusion:**

The proposed model evolution method provides a practical way for the KBP to enhance its prediction ability with minimal human intervene. This highly refined prediction model can better guide KBP in improving the consistency and quality of the VMAT plans.

## Introduction

Volumetric modulated arc therapy (VMAT) followed by intracavitary brachytherapy has become one of major treatment modalities for cervical cancer [1–4]. However, developing an appropriate VMAT plan presents a real challenge, since inverse VMAT planning in essence is still a trial-and-error procedure. The planner has to manually set the starting optimization objectives for the tumor target as well as for each organ of interest, which needs to take into account the patient anatomy, the linac performance, the prescription doses and the organ dose tolerance limits. This makes VMAT planning operator- and experience-dependent, as too “easy” objectives may lead to suboptimal plan while too hard objectives may cause sub-optimal trade-offs. Several authors have reported some head & neck and prostate cases suffering from over irradiation to the organs at risk (OAR) due to suboptimal treatment plans [5–7]. To address this issue, knowledge-based planning (KBP) arouses growing interest, which utilizes the prior knowledge to predict what kind of dose distribution is achievable and hence automatically generates the patient-specific optimization objectives for each OAR according to the estimated dose volume histograms (DVHs). Various KBP methods have been developed [8–14] and among them, RapidPlan™ is the first commercial software that has been put into clinical use. Previously published works have demonstrated its usefulness in improving plan quality and planning efficiency for tumors in head & neck, prostate and rectum [15–19].

One of major concerns about the use of KBP is the quality of the plan database, which may determine the degree of accuracy that a prediction model can offer. It has already been revealed that current estimated results can only fulfil the “clinical acceptable” criteria rather than “optimal” or “near optimal” standards, due to the fact that the database plans may not all possess optimal dose distributions [5, 9]. Some researches tried to re-optimize each of prior plans by a group of experts to guarantee a high quality [20–22]. This is tremendously labor intensive and time consuming, especially for cases where a large number of training samples are used. To improve the predictive accuracy more efficiently, Appenzoller et al. introduced a refined method to take the estimated DVHs as a reference to exclude suboptimal plans from the training cohort and repeat the modeling process on the remaining training dataset [11]. Wang et al. demonstrated that both the prediction model and its constituent plans were able to be significantly improved after two runs of closed loop refinements [22]. More recently, a refined model has been applied in an ongoing multi-institutional clinical trial as a quality assurance tool, highlighting its great potential for accurate DVH prediction [21]. Nevertheless, as it has been supposed that the quality of the database can be improved over time by using the KBP method [5, 9, 13], this may suggest that the prediction model should also be retrained on a regular basis to ensure its predictive accuracy. Therefore, it was expected to develop a progressive training strategy striving to create a highly refined KBP model with minimum human intervene.

Another issue often encountered in model generation is how many patient plans are recommended to build a particular prediction model. The manufacturer recommended that the minimum number of plans required for RapidPlan model creation was 20, but they emphasized that adding additional plans would usually help create a more robust model [23]. A newly published research concluded that the minimum required sample size needed to accurately train KBP models for prostate cancer depends on the specific model and endpoint to be predicted, and a sample size greater than 75 was recommended to train the KBP models [24]. Hence it is of primary importance to determine a propriate number of training samples in establishing the prediction model for cervical cancer to maintain its accuracy and robustness.

In this study, we present our experience in the application of KBP in VMAT treatment of cervical cancer with special attention to the above issues. A highly refined DVH prediction model was built for VMAT treatment of cervical cancer, which underwent a total of six runs of refining through progressive training until the training set size increased up to 100 cases. The proposed model evolution method was assessed in 35 new cervical cancer cases. The reliability and efficiency of KBP using this highly refined model in improving the consistency and quality of the VMAT plans were analyzed.

## Methods and materials

### Database

A total of 100 patients with stage IA-IVB cervical cancer treated by pelvic VMAT were retrospectively reviewed. All patients were immobilized in the supine position. The CT images were acquired by a CT simulator using 3 mm slice and 3 mm spacing. The gross target volume (GTV) included all grossly enlarged lymph nodes with a short diameter of ≥ 1 cm and regional metastatic lymph nodes on imaging findings or as determined by PET/CT findings. The clinical target volume (CTV) included the cervix, whole uterus, parametrium, upper part of the vagina, and pelvic lymphatic drainage area (common, internal, and external iliac; obturator; and presacral). Inguinal lymph nodes were included if lower one-third vaginal involvement was observed. In patients with common iliac metastatic lymph nodes, para-aortic irradiation was administered. The planning gross target volume (PGTVnd) was generated by adding a 5-mm margin to the GTV and the planning clinical target volume (PCTV) was generated by adding a 6-mm margin to the CTV in all orientations, except for the anterior direction where a 10-mm margin was used. Dual-arc VMAT plans were designed by using Varian Eclipse treatment planning system (Varian Medical Systems, Palo Alto, CA), including two coplanar full arcs with gantry rotating counterclockwise from 179° to 181° and clockwise from 181° to 179°. Dose prescription was set to be 60 Gy in 25 fractions to the PGTVnd and 45 Gy in 25 fractions to the PCTV. The planning goals for tumor targets and dose constraints for the OARs were detailed in Table 1. Recent follow-up indicated that all patients were proved to have favorable prognoses with neither severe late toxicity nor treatment failure (local recurrence/distant metastasis).

**Table 1:** Plan quality evaluation criteria. The planning goals for tumor target and dose constraints for OARs were listed

	Criteria		Quality scores
	Acceptable	Excellent	
PGTVnd	V_60Gy_ (%) ≥ 97	V_60Gy_ (%) = 100	4.8/8
	V_66Gy_ (%) ≤ 10	V_66Gy_ (%) ≤ 5	4.8/8
	D_min_ (Gy) ≥ 54.6	D_min_ (Gy) ≥ 55.8	3/5
	CI (0–1):	As high as possible	0–2
	HI (0–1):	As low as possible	0–2
PCTV	V_45Gy_ (%) ≥ 97	V_45Gy_ (%) = 100	4.8/8
	V_49.5 Gy_ (%) ≤ 20	V_49.5 Gy_ (%) ≤ 10	4.8/8
	D_min_ (Gy) ≥ 40.95	D_min_ (Gy) ≥ 41.85	3/5
	CI (0–1):	As high as possible	0–2
	HI (0–1):	As low as possible	0–2
Spinal cord	D_0.03 cc_ (Gy) ≤ 45	D_0.03 cc_ (Gy) ≤ 40	6/10
Bladder	D_35.0%_ (Gy) ≤ 50	D_35.0%_ (Gy) ≤ 45	6/10
Rectum	D_60.0%_(Gy) ≤ 45	D_60.0%_(Gy) ≤ 40	6/10
Kidney (L)	V_18Gy_ (%) ≤ 32	V_18Gy_ (%) ≤ 20	3/5
Kidney (R)	V_18Gy_ (%) ≤ 32	V_18Gy_ (%) ≤ 20	3/5
Femoral head (L)	V_35Gy_ (%) ≤ 50	V_35Gy_ (%) ≤ 15	1.8/3
	D_0.03 cc_ (Gy) ≤ 65	D_0.03 cc_ (Gy) ≤ 50	1.2/2
Femoral head (R)	V_35Gy_ (%) ≤ 50	V_35Gy_ (%) ≤ 15	1.8/3
	D_0.03 cc_ (Gy) ≤ 65	D_0.03 cc_ (Gy) ≤ 50	1.2/2
Total score	/	/	100

### Model building and evolution

The prediction model was automatically generated for pelvic organs of interest, specific for each OAR, based on the principle of parameterization of the structure set and dose matrices for the prior plans in the training set. The built-in proprietary algorithm for the RapidPlan™ (version 13.5, Varian Medical Systems, Palo Alto, CA) is largely inspired by the methodology described by Yuan et al. [25].

**Fig. 1:**
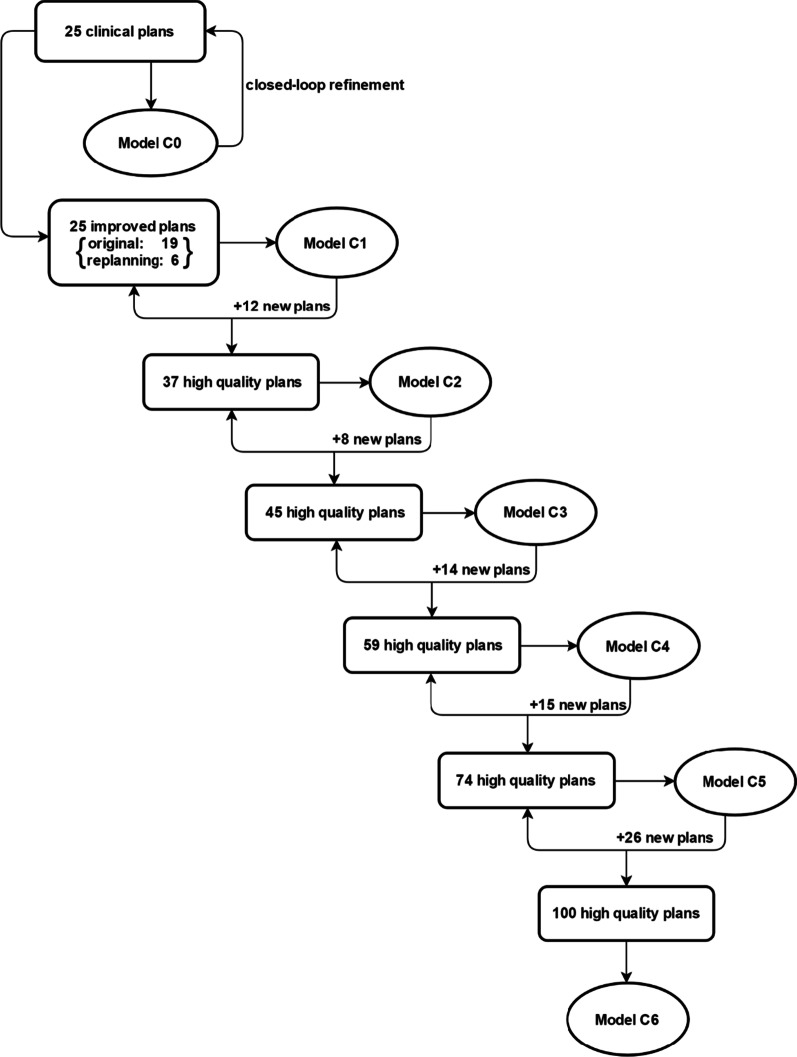
The diagram of the proposed model evolution process. The prediction model functions as a self-checking tool, ensuring that only new plans with quality higher than past plans can be added to the training dataset. The developed model underwent a total of six runs of refinements with training set size increased from initial 25 plans up to 100 cases

In this study, an in-house model evolution strategy was developed to enhance the prediction ability of the model by progressively upgrading the database with new higher-quality plans and re-training the model. The developed model was initially built using 25 clinically approved VMAT plans for cervical cancer (model C_0_), which was the minimum number of treatment plans suggested by the product specialist. A closed-loop refinement process was conducted subsequently, in which relatively suboptimal plans were identified by comparing estimated DVHs with planned DVHs. Unlike previous studies [5, 9], these suboptimal plans were not excluded from the database, but were rejoined to the training dataset after they were re-optimized under the guidance of estimated DVHs to further spare the OARs. This resulted in a refined model C_1_, preliminarily applied in clinic: (1) To guide the planning/re-planning process with better OAR sparing achieved; (2) To be a self-checking tool to identify the quality of the plan. By this means, VMAT plans with quality superior to the prediction were screened out and were added to the database to re-train the model on a monthly basis. Within the past 6 months, the developed model underwent five runs of refinement, generating model C_2_-C_6_, with training set size increased up to 100 cases. The detailed diagram of our model evolution process was illustrated in Fig. 1.

### Dosimetric evaluation

The proposed model evolution method was assessed in 35 new cervical cancer cases. For comparison purpose, three kinds of VMAT plans were developed for each patient. (1) Automatic plan (AP): automatically created by only one click at the “optimization” button with no other human intervention; (2) Manual plan (MP): designed independently by a qualified planner in the traditional trial-and-error way; (3) Clinically approved plan (CP): created based on AP, but unlike AP, possible manual adjustments are permitted thereafter. The CPs were regarded as the reference standard in our plan comparison.

The prediction ability of the refined models of different stages was evaluated by comparing the estimated DVHs with the actual dose distributions finally achieved (i.e., DVHs derived from the CP, which was herein taken as the reference). This was done by assessing the degree of approximation between the predicted values and the reference values at given dosimetric endpoints. In consequence, the difference (in the absolute value) between the estimated dose and the reference dose of every model for a given OAR was calculated, and was ranked from small to large, with 6 points for the first, 5 points for the second, 4 points for the third, and so on. A total of 35 cases were evaluated and the average scores of various models were obtained for each OAR. The full mark of this investigation was 6 points. The introduction of such a scoring method is mainly to minimize the impact of individual cases on the global results.

To evaluate the usefulness of such a highly refined model in VMAT planning, a dosimetric comparison was conducted between APs generated by using model C_1_ and model C_6_, respectively. The CPs generated based on model C_6_ were also compared with the MPs with respect to the target coverage, the OAR sparing and the planning time. The effectiveness of KBP with this highly refined model in improving the consistency and quality of the VMAT plans were analyzed.

The dosimetric indices herein used for target dose evaluation include the dose coverage, the CI and HI. The conformity index (CI) [26] was calculated by:

$$Conformity \, Index (CI)=\left(\frac{{V}_{Tref}}{{V}_{T}}\right)\times \left(\frac{{V}_{Tref}}{{V}_{ref}}\right)$$

where V_*Tref*_ refers to the volume of the target covered by the reference isodose (here 95% isodose), V_*T*_ was the target volume, and V_*ref*_ was the volume of the reference isodose (i.e., 98% isodose).

The homogeneity index (HI) was defined as follows:

$$Homogeneity\, Index (HI)=\frac{{D}_{2\%}-{D}_{98\%}}{{D}_{50\%}}$$

where D_x%_ refers to the absorbed dose received by x% of the target volume [27].

The dosimetric indices to OARs were selected according to their radiobiological properties. The average dose (D_mean_) was computed for parallel organs, while the maximum dose (D_max_) was recorded for serial organs like spinal cord. Other dosimetric indices collected include: V_18 Gy_ for kidney (L/R) and V_30 Gy_, V_40 Gy_ and V_50 Gy_ for bladder, rectum, and femoral head (L/R).

To quantify the difference between plans, an assessing tool, namely Plan Quality Metric (PQM), was introduced [28, 29]. The penalty points were assigned to the tumor target and each OAR, according to the priority of dose optimization objectives. The built-in dosimetric endpoints were determined with reference to our institutional protocols and the RTOG 0921 guideline [30]. The scoring details were listed in Table 1.

### Statistical analysis

All statistical analyses were performed with SPSS software (version 20, SPSS Inc, Chicago, IL). The analyses of variance (ANOVA) were applied when normality (and homogeneity of variance) assumptions are satisfied. Otherwise, Wilcoxon Signed Rank test will be used. The statistically significant level was set as 0.05.

## Results

**Fig. 2:**
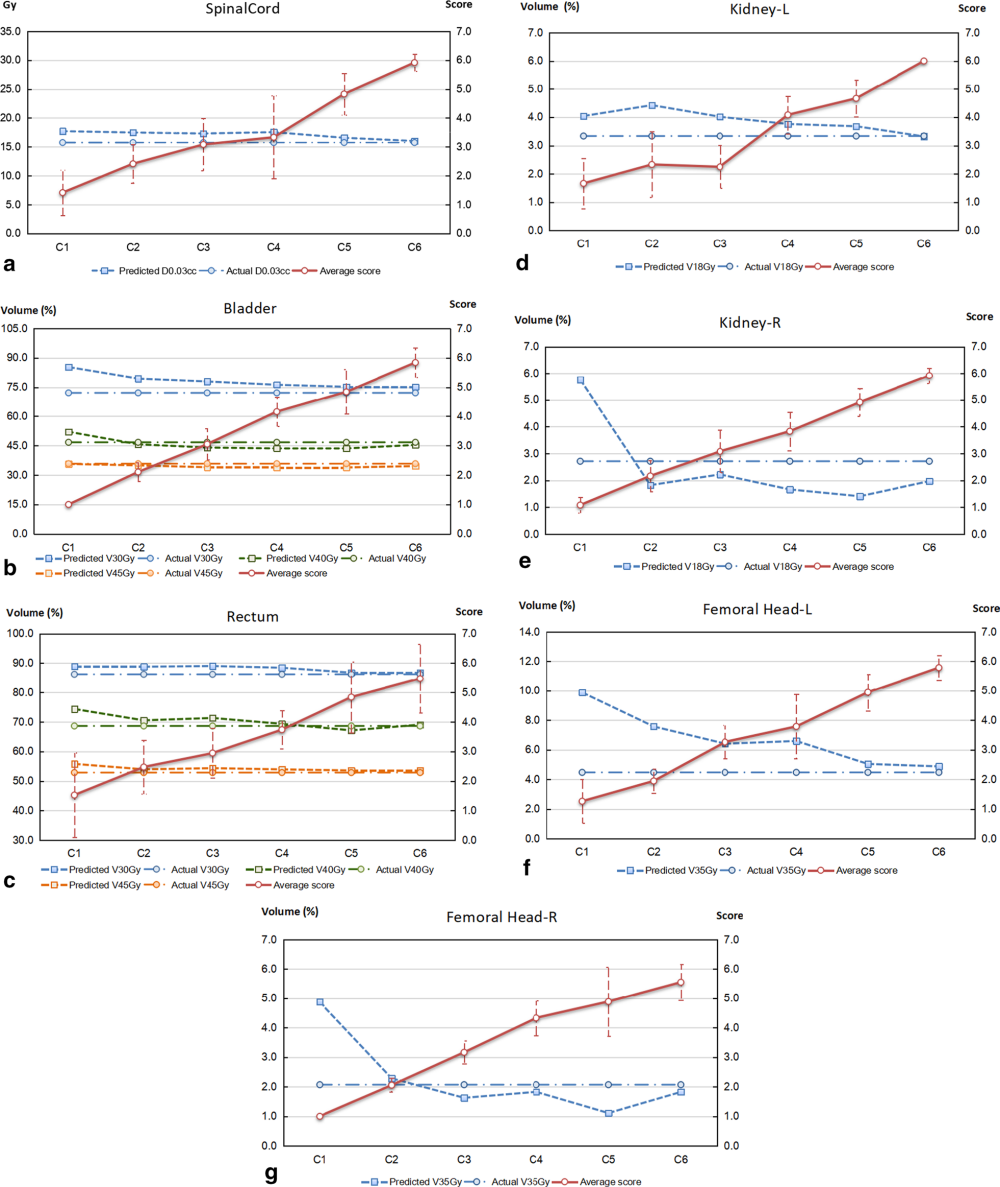
Comparison of the estimated DVHs derived from the refined model of different runs of training with the planning DVHs finally achieved for 35 cervical cancer cases. a, Spinal Cord; b, Bladder; c, Rectum; d, Kindey (L); e, Kidney (R); f, Femoral head (L); and g, Femoral head (R). The left coordinate system corresponds to the predicted key dosimetric values and the right coordinate system corresponds to the score results

Figure 2 plotted the predicted doses, the actual doses, and the scores for different models of a certain organ of 35 cervical cancer patients. There is a tendency that the predictive accuracy was reinforced with the increased number of refinements, in terms of the degree of approximation of the predicted doses to the actual values. This can be more clearly seen in the scoring curve, which minimize the impact of individual cases on the global results by introducing the weighted scores. The predictive outcomes of model C_1_ were relatively poor, most of which were ranked at the bottom and got the lowest score. Model C_5_ obtained a score approximate to 5 points in most cases, while model C_6_ provided the best estimate to the actual doses among the refined models of different stages. The associated scores for model C_6_ were all above 5.5 points for various tested OARs (The full mark is 6 points). It seems that the prediction ability approaches the limits of current planning skills (i.e., best effort plan) after five to six runs of re-training, when the training samples increases up to about 75 to 100 cases.

The refined model C_1_ and C_6_ were applied in automatic KBP for cervical cancer. Compared with APs created by model C_1_ (AP-1), APs created by model C_6_ (AP-6) shows advantages in dealing with the trade-offs between the target coverage and the dose to the OAR (Table 2). The proportion of AP-6 that can directly satisfy the clinical requirements without any manual revision was 22/35, while that of AP-1 was 16/35. The plan quality scoring gave the average values of 85.61 ± 6.78 and 83.92 ± 6.86 for AP-6 and AP-1 (p = 0.013).

**Table 2:** Dosimetric comparison of AP-1 vs. AP-6. The statistical results between AP-1 scores and AP-6 scores were also given

		AP-1		AP-6		*p* value
		Results	Scores	Results	Scores	
PGTVnd	V_60Gy_ (%)	94.19 ± 8.76	4.11 ± 2.77	96.86 ± 4.91	5.46 ± 2.73	< 0.001
	V_66Gy_ (%)	0.00 ± 0.00	8.00 ± 0.00	0.00 ± 0.00	8.00 ± 0.00	/
	D_min_ (Gy)	58.16 ± 2.59	4.76 ± 0.92	58.52 ± 1.60	4.80 ± 0.77	0.159
	CI	0.62 ± 0.11	1.24 ± 0.21	0.58 ± 0.11	1.16 ± 0.22	< 0.001
	HI	0.05 ± 0.02	1.90 ± 0.04	0.05 ± 0.02	1.89 ± 0.03	0.240
PCTV	V_45Gy_ (%)	95.96 ± 6.94	4.79 ± 1.79	98.55 ± 3.16	6.81 ± 1.33	< 0.001
	V_49.5 Gy_ (%)	13.67 ± 6.42	6.71 ± 1.92	18.90 ± 6.83	5.11 ± 2.05	< 0.001
	D_min_ (Gy)	36.72 ± 10.69	2.23 ± 2.03	38.01 ± 10.79	3.05 ± 2.17	0.008
	CI	0.84 ± 0.03	1.68 ± 0.06	0.82 ± 0.02	1.65 ± 0.04	< 0.001
	HI	0.32 ± 0.06	1.37 ± 0.12	0.30 ± 0.06	1.40 ± 0.11	0.095
Spinal cord	D_0.03 cc_ (Gy)	14.13 ± 12.49	10.00 ± 0.00	14.38 ± 13.44	10.00 ± 0.00	/
Bladder	D_35%_ (Gy)	42.84 ± 8.13	9.50 ± 0.61	43.54 ± 8.10	9.18 ± 0.88	< 0.001
Rectum	D_60.0%_(Gy)	41.54 ± 7.47	7.72 ± 1.44	42.43 ± 7.63	7.40 ± 1.65	< 0.001
Kidney (L)	V_18Gy_ (%)	3.05 ± 4.71	5.00 ± 0.00	2.90 ± 4.60	5.00 ± 0.00	/
Kidney (R)	V_18Gy_ (%)	3.16 ± 7.19	4.91 ± 0.53	2.32 ± 3.88	5.00 ± 0.00	0.324
Femoral head (L)	V_35Gy_ (%)	7.80 ± 3.80	3.00 ± 0.02	5.59 ± 3.29	3.00 ± 0.00	0.211
	D_0.03 cc_ (Gy)	40.95 ± 7.47	2.00 ± 0.00	41.12 ± 7.61	2.00 ± 0.00	/
Femoral head (R)	V_35Gy_ (%)	5.55 ± 4.79	2.96 ± 0.17	3.70 ± 4.96	2.96 ± 0.18	0.879
	D_0.03 cc_ (Gy)	38.98 ± 7.74	2.00 ± 0.00	39.48 ± 7.63	2.00 ± 0.00	/
Total score			83.92 ± 6.86		85.61 ± 6.78	0.013

AP refers to the fully automatic plan, which was created by only one click at the “optimization” button with no other human intervention. AP-1 and AP-6 were fully automatic plans generated by using model C_1_ and model C_6_, respectively

The dosimetric results of CPs vs. MPs were given in Table 3. It was shown that both sets of VMAT plans achieved the dose coverage of V_60Gy_ higher than 99% for PGTVnd and PCTV. Compared with MPs, CPs exhibited lower V_110%_ (*p* < 0.001) and better CI (*p* = 0.001) for PCTV at a slight sacrifice of target dose coverage (*p* = 0.011) and minimal dose D_min_ (*p* = 0.002). However, The D_min_ was all greater than 93% of the prescription dose for both kinds of treatment plans. The average plan quality scores for tumor target (PGTVnd plus PCTV) were 43.39 ± 4.04 and 42.23 ± 3.47 for CP and MP (*p* = 0.011) (Table 4).

**Table 3:** Dosimetric results for two kinds of VMAT plans. The corresponding statistical analyses were also included

		CP	MP	*p* value
PGTVnd	V_60Gy_ (%)	99.36 ± 0.59	99.40 ± 0.71	0.785
	V_66Gy_ (%)	0.15 ± 0.53	0.08 ± 0.35	0.095
	D_min_(Gy)	59.12 ± 1.01	59.35 ± 0.73	0.131
	CI	0.54 ± 0.11	0.54 ± 0.10	0.717
	HI	0.05 ± 0.01	0.05 ± 0.01	0.444
PCTV	V_45Gy_ (%)	99.48 ± 0.58	99.73 ± 0.20	0.011
	V_49.5 Gy_ (%)	16.04 ± 9.50	24.43 ± 12.16	< 0.001
	D_min_(Gy)	38.44 ± 10.87	39.49 ± 11.10	0.002
	CI	0.81 ± 0.03	0.79 ± 0.03	0.001
	HI	0.30 ± 0.06	0.30 ± 0.06	0.875
Spinal cord	D_0.03 cc_(Gy)	14.72 ± 13.67	15.42 ± 14.28	0.019
Bladder	D_mean_(cGy)	3791.45 ± 704.91	4076.11 ± 743.14	< 0.001
	D_35%_(Gy)	43.79 ± 8.18	44.66 ± 7.95	0.004
	V_30_(%)	75.92 ± 16.59	89.66 ± 18.34	< 0.001
	V_40_(%)	50.20 ± 15.19	66.03 ± 17.42	< 0.001
	V_50_(%)	3.67 ± 3.40	2.27 ± 2.72	0.003
Rectum	D_mean_(cGy)	4189.89 ± 751.59	4301.27 ± 759.75	< 0.001
	D_60%_(Gy)	43.09 ± 7.82	44.31 ± 7.82	0.001
	V_30_(%)	90.46 ± 16.36	93.09 ± 16.73	< 0.001
	V_40_(%)	75.40 ± 16.40	84.76 ± 16.64	< 0.001
	V_50_(%)	1.87 ± 3.79	1.24 ± 3.55	0.277
Kidney-L	D_mean_(cGy)	427.02 ± 416.72	488.35 ± 486.71	0.001
	V_18_(%)	3.22 ± 5.05	4.73 ± 7.19	0.019
Kidney-R	D_mean_(cGy)	472.90 ± 390.95	575.53 ± 483.65	< 0.001
	V_18_(%)	2.65 ± 4.53	6.42 ± 7.10	< 0.001
Femoral-L	D_mean_ (cGy)	1667.98 ± 438.95	2164.23 ± 541.71	< 0.001
	D_0.03 cc_(Gy)	41.23 ± 7.68	43.94 ± 7.76	< 0.001
	V_30_(%)	12.82 ± 6.76	26.61 ± 14.25	< 0.001
	V_35_(%)	5.91 ± 3.58	15.94 ± 8.77	< 0.001
	V_40_(%)	1.25 ± 1.54	6.24 ± 3.81	< 0.001
	V_50_(%)	/	/	/
Femoral-R	D_mean_(cGy)	1551.25 ± 432.40	2117.46 ± 571.05	< 0.001
	D_0.03cc_(Gy)	39.70 ± 7.73	43.43 ± 7.84	< 0.001
	V_30_(%)	9.32 ± 8.71	24.51 ± 16.05	< 0.001
	V_35_(%)	4.01 ± 5.41	14.91 ± 13.59	< 0.001
	V_40_(%)	0.89 ± 2.04	6.25 ± 8.76	< 0.001
	V_50_(%)	/	/	/

MP refers to the manual plan. CP refers to the clinically approved plan, which was created based on knowledge-based planning with possible manual adjustments thereafter. The CP was taken as our reference standard in this study

**Table 4:** Plan quality scores for CPs and MPs. The corresponding statistical analyses were included

		CP	MP	*p* value
PGTVnd	V_60Gy_ (%)	7.31 ± 0.63	7.36 ± 0.76	0.784
	V_66Gy_ (%)	8.00 ± 0.00	8.00 ± 0.00	/
	D_min_ (Gy)	4.98 ± 0.12	5.00 ± 0.00	0.324
	CI	1.07 ± 0.21	1.08 ± 0.20	0.679
	HI	1.90 ± 0.03	1.90 ± 0.02	0.473
PCTV	V_45Gy_ (%)	7.44 ± 0.61	7.71 ± 0.21	0.011
	V_49.5 Gy_ (%)	6.04 ± 2.71	4.04 ± 2.56	< 0.001
	D_min_ (Gy)	3.62 ± 1.97	4.17 ± 1.60	0.025
	CI	1.62 ± 0.05	1.57 ± 0.07	0.001
	HI	1.40 ± 0.12	1.40 ± 0.13	0.936
Spinal cord	D_0.03 cc_ (Gy)	10.00 ± 0.00	10.00 ± 0.00	/
Bladder	D_35%_ (Gy)	9.03 ± 0.97	8.90 ± 0.85	0.102
Rectum	D_60%_(Gy)	6.60 ± 1.95	5.62 ± 1.40	< 0.001
Kidney (L)	V_18Gy_ (%)	5.00 ± 0.00	4.97 ± 0.18	0.315
Kidney (R)	V_18Gy_ (%)	5.00 ± 0.00	4.98 ± 0.09	0.324
Femoral head (L)	V_35Gy_ (%)	3.00 ± 0.01	2.89 ± 0.24	0.009
	D_0.03 cc_ (Gy)	2.00 ± 0.00	2.00 ± 0.00	/
Femoral head (R)	V_35Gy_ (%)	2.96 ± 0.18	2.85 ± 0.45	0.081
	D_0.03 cc_ (Gy)	2.00 ± 0.00	2.00 ± 0.00	/
Total score		89.02 ± 4.83	86.48 ± 3.92	< 0.001

MP refers to the manual plan. CP refers to the clinically approved plan, which was created based on knowledge-based planning with possible manual adjustments thereafter

As for the radiation dose to OARs, CPs significantly reduced almost all the dosimetric indices except for the bladder V_50Gy_ (%) and the rectum V_50Gy_ (%), compared with MPs (Table 3). The overall quality assessment gave the mean scores of 89.02 ± 4.83 and 86.48 ± 3.92, respectively, for CPs and MPs (*p* < 0.001) (Table 4).

## Discussion

Although pelvic VMAT has been an increasingly used technique for treatment of cervical cancer, designing an appropriate VMAT plan remains a challenge. The major difficulty lies in the fact that the planner usually does not know what kind of dose distribution is achievable for each OAR. Due to limited planning time, especially in some busy centers, the planner may not have enough chance to repeatedly adjust the dose distributions to explore whether there are better results. This tends to lead to some suboptimal plans. It was shown by us and others [31, 32] that there were quite a few clinical plans that have room for improvement. Therefore, it has become a top priority to develop a way to improve the consistency and quality of the VMAT plans.

The KBP model can provide the estimated DVHs based on the prior knowledge, helping direct the planner’s efforts towards an achievable high-quality plan. It was observed that the prediction ability was enhanced with the increased number of refinements in terms of OAR sparing for most OARs. This may result from the joint actions of the increased number of training samples and the improved quality of the plan database. Currently, there are few studies related to the required training samples size for a particular KBP model. The manufacturer specialists suggested that the minimum number of plans required for RapidPlan model creation was 20, but they also emphasized that adding additional plans would usually help create a more robust model [23]. Meanwhile, a newly published research discussed that although only 20 samples were needed to predict the rectum DVH, a sample size greater than 75 was recommended to train the KBP model [24]. This is why we started training from 25 samples and finally increased the sample size up to 100 cases. Our experiments proved once again the advantages of large training sample size in establishing the prediction model. By continuously updating the database with new plans of higher quality than before, the quality of the database was improved over time in a systematic way, which had interactive impact on the KBP model. The prediction model experiencing several runs of progressive training was found to provide better estimates for the final dose distribution.

With enhanced prediction ability, the highly refined model has shown its advantage in capturing actual clinical practices during the knowledge-based VMAT planning of cervical cancer. More than 60% of AP-6 plans can be directly approved for clinical treatment. The primary reason for the failure of automatic planning is the insufficient coverage of PCTV by the prescription dose in the overlapping region of PCTV to rectum and PCTV to bladder. There is a tendency for pelvic radiotherapy in our practice that the high dose coverage of tumor target area will be improved preferentially to ensure local tumor control when the radiation doses of critical organs do not exceed the dose tolerance limits. However, the RapidPlan takes the lower bound of the DVH estimate range as the optimization objectives by default with attempt to maximize OAR sparing. This, on the other hand, may lead to underdose of the adjacent tumor target. Adding a 3-mm ring structure outside the PCTV to allow for the high dose fall-off helps improve the success rate of automatic planning. More research is warranted.

Compared with the conventional trial-and-error planning method, our results demonstrated that the novel KBP method could enhance the quality of treatment planning in term of better OAR protection. Radiation induced acute and chronic toxicities, including small bowel obstruction, enteritis, proctitis, and radiation cystitis are serious issue of concern directly related to the quality of life. It has been reported that the incident rates of grade 3 or higher complications in 83 patients treated with pelvic IMRT plus high dose rate brachytherapy are 2.4% and 3.6% for the rectum and the bladder, respectively [1]. By applying KBP, the dosimetric indices of the rectum and the bladder, such as D_mean_, V_40Gy_ and V_30Gy_, were all significantly reduced (*p* < 0.001) under the condition of approximately similar target dose distributions. This may contribute to the fact that the incidence of late toxicities at our institution appeared to be lower than those reported in previous studies [3]. Moreover, the KBP method was found to help standardize the patient treatment, making treatment results less operator- and experience-dependent. In dosimetric comparisons, even CPs designed by a junior planner can achieve dose distributions comparable to MPs. Notably, the time spent for a KBP plan is much lower than that for a manual plan, even if manual revision is required.

## Conclusion

The proposed model evolution method not only utilizes the KBP model to guide the planning process, but also takes it as a self-checking tool to identify high quality plans, providing a practical way to enhance the prediction ability with minimal human intervene. It has proved to our satisfaction that this highly refined prediction model can better guide KBP in improving the consistency and quality of the VMAT plans. The method described here was universal and can be used for some other cancer sites. However, in order to satisfy the diverse needs of clinical practice, it is recommended that each unit establishes its own model using this refinement method.

## Data Availability

The datasets are backed up on the Research Data Deposit (RDD Number: RDDA2020001629, https://www.researchdata.org.cn) and are available upon reasonable request.
